# Case Report: A Novel Non-Reciprocal ALK Fusion: ALK-GCA and EML4-ALK Were Identified in Lung Adenocarcinoma, Which May Respond to Alectinib Adjuvant-Targeted Therapy

**DOI:** 10.3389/fonc.2021.782682

**Published:** 2022-01-05

**Authors:** Xiaoqian Zhai, Qiang Wu, Dan Pu, Liyuan Yin, Weiya Wang, Daxing Zhu, Feng Xu

**Affiliations:** ^1^ Lung Cancer Center, West China Hospital, Sichuan University, Chengdu, China; ^2^ Pathology Department, West China Hospital, Sichuan University, Chengdu, China

**Keywords:** ALK fusion, ALK-GCA and EML4-ALK, stage IIIB-N2 NSCLC, alectinib, adjuvant targeted therapy

## Abstract

Anaplastic lymphoma kinase (ALK)-positive non-small-cell lung cancers (NSCLCs) have favorable and impressive response to ALK tyrosine kinase inhibitors (TKIs). However, ALK rearrangement had approximately 90 distinct fusion partners. Patients with different ALK fusions might have distinct responses to different-generation ALK-TKIs. In this case report, we identified a novel non-reciprocal ALK fusion: ALK-grancalcin (GCA) (A19: intragenic) and EML4-ALK (E20: A20) by next-generation sequencing (NGS) in a male lung adenocarcinoma patient who was staged as IIIB-N2 after surgery. After a multidisciplinary discussion, the patient received alectinib adjuvant targeted therapy and postoperative radiotherapy (PORT). He is currently in good condition, and disease-free survival (DFS) has been 20 months so far, which has been longer than the median survival time of IIIB NSCLC patients. Our study extended the spectrum of ALK fusion partners in ALK + NSCLC, and we reported a new ALK fusion: ALK-GCA and EML4-ALK and its sensitivity to alectinib firstly in lung cancer. It is vital for clinicians to detect fusion mutations of patients and report timely the newfound fusions and their response to guide treatment.

## Background

Approximately 83% of lung cancers are classified as non-small-cell lung cancer (NSCLC) (1), 5%–7% of which harbor oncogenic anaplastic lymphoma kinase (ALK) fusion and are defined as ALK-positive NSCLC at diagnosis (2). ALK tyrosine kinase inhibitors (TKIs), including crizotinib, alectinib, and ceritinib, demonstrated outstanding efficacy and tolerability for the treatment of ALK-rearranged NSCLC (3–5). In particular, alectinib which has higher blood–brain barrier permeability and lower toxicities, is currently used widely in the first line of *ALK+*NSCLC in the metastatic setting (5).

Despite favorable results, ALK-rearranged NSCLC is a heterogeneous disease. Patients with different ALK fusions have distinct responses to ALK-TKIs (6). Apart from the classical EML4-ALK fusion, ALK rearrangement had approximately 90 distinct fusion partners, like LTBP1 and STRN OFCC1 (6–9). Some of the ALK rearrangements presented crizotinib and alectinib resistance primarily. ALK rearrangement could be mainly categorized into three types: 3′-ALK fusion alone, 5′-ALK fusion alone, and non-reciprocal/reciprocal ALK (10). A research reported that non-reciprocal ALK fusion partners were poor predictive markers and would have an impact on the ALK-TKI response with shorter PFS (10). Thus, in terms of this heterogeneity of ALK-positive NSCLC, it is vital for clinicians to detect fusion mutations of patients and report timely the newfound fusions and their response to guide treatment. Herein, we report a new non-reciprocal ALK fusion: ALK-GCA (A19: intragenic) and EML4-ALK (E20: A20) detected by next-generation sequencing (NGS) for the first time from a patient with lung adenocarcinoma.

## Case Presentation

**Figure 1:**
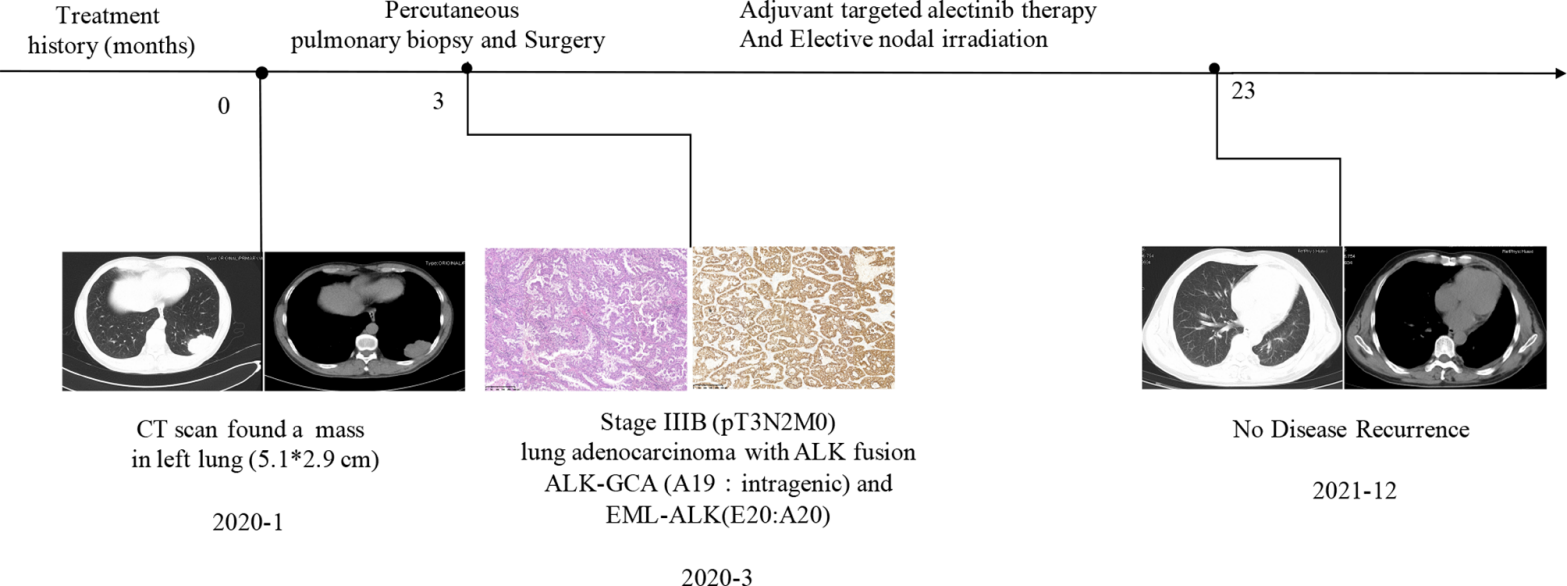
The clinical treatment history of the patient. Chest computed tomography (CT) and histological findings were presented. HE staining showed lung adenocarcinoma. Immunochemistry results suggested non-reciprocal ALK fusion: ALK-GCA (A19: intragenic) and EML4-ALK (E20: A20) expressed significantly at the protein level.

**Figure 2:**
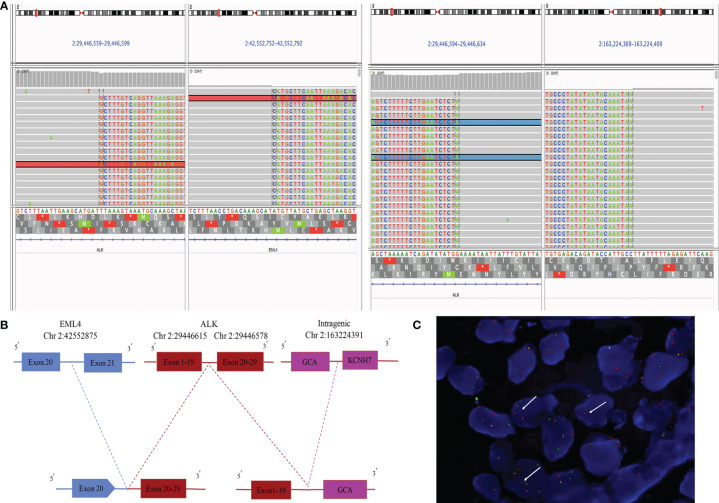
Next-generation sequencing and FISH results of surgical specimen of the patient. **(A)** ALK-GCA (A19: intragenic) and EML4-ALK (E20: A20) ALK rearrangement visualized using the Integrative Genomics Viewer (IGV). **(B)** Diagram depicting the non-reciprocal ALK fusion: ALK-GCA (A19: intragenic) and EML4-ALK (E20: A20). **(C)** ALK gene rearrangement positive signal (one green and one red, by white arrow) (*1,000).

A 55-year-old male Chinese patient, without a smoking history and family history of cancer, complained of left chest pain subsequent to a fall and presented to our hospitals. Physical examination showed he was tender to palpation at the left 7–10 rib. Chest computed tomography (CT) scan revealed a lung mass with a diameter of about 5.1 cm in the left lower lobe and 7–10 rib fracture in January 2020 (**Figure 1**). Then, percutaneous pulmonary biopsy was performed and found lung adenocarcinoma. For further treatment, he received pulmonary lobectomy in March 2020, with a pathologic diagnosis of stage IIIB (pT3N2M0) moderately and poorly differentiated invasive lung adenocarcinoma (acinar type, micropapillary pattern, solid form) (**Figure 1**). Then, immunochemistry (IHC-ventana D5F3) of the tumor specimen was performed and the results showed a significant ALK fusion protein expression (**Figure 1**). Furthermore, we conducted fluorescence *in situ* hybridization (FISH) (11) and confirmed the positive ALK gene break using commercially available probes (GSP ALK, Anbiping Pharmaceutical Technology Co., Ltd.) (**Figure 2C**). In addition, as previously described (12), the genomic profiles of surgical specimens were assessed by performing capture-based targeted deep sequencing using the Lung Core panel (Burning Rock Biotech, Guangzhou, China), which covers the whole exons of 68 lung cancer-related genes and spans 345 kb of the human genome (**Supplementary Material 2**). DNA quality and size were assessed by high-sensitivity DNA assay using a bioanalyzer. All indexed samples were sequenced on a NextSeq 500 system (Illumina, Inc.) with pair-end reads with a target sequencing depth of 1,000* for tissue samples. The NGS identified a non-reciprocal ALK fusion: ALK-GCA (A19: intragenic) (abundance: 17.18%) and EML4-ALK (E20: A20) (abundance: 14.47%) (**Figure 2A**). After a multidisciplinary discussion, alectinib as adjuvant targeted therapy was administrated for him to reduce the risk of tumor recurrence. In addition, concerning that the patient had mediastinal lymph node metastasis, then postoperative radiotherapy (PORT) in terms of elective nodal irradiation (ENI) with a total dose of 50 Gy in 25 fractions was performed in October 2020. The toxicities of combined therapy for the patient were tolerable. The patient is currently in good condition, and disease-free survival (DFS) has been 20 months so far. We will continue to follow up and dynamically monitor the changes of these two ALK fusions in the future treatment process.

## Discussion

The non-reciprocal ALK translocation was defined firstly in *Journal of Thoracic Oncology* by Yongchang Zhang et al., as harboring concurrent ALK fusions with at least one 3′-ALK fusion and one 5′-ALK when compared to 3′-ALK alone fusion. We showed a schematic diagram of non-reciprocal ALK translocation in **Supplementary Figure 1**. Therefore, ALK-GCA (A19: intragenic) as 5′-ALK and EML4-ALK (E20: A20) as 3′-ALK in our case were defined together as a non-reciprocal ALK fusion (**Figure 2A**). This fusion was the GCA intergenic region rearranged to exons 1–19 of the ALK gene, while the EML4 broken gene fused to the exons 20–29 of the ALK gene, which contain the ALK kinase domain (**Figure 2B**). Based on sequence analysis, we speculated that two ALK fusions may be harbored by the same tumor clone. However, in fact, we could not be completely sure that the two ALK fusions are from the same clone of tumor cells, or two different populations in the adenocarcinoma. There is a limitation that the FISH results in our hospital could only prove that the ALK gene has been broken and rearranged in a cell. It could not confirm whether GCA-ALK and EML4-ALK exist in one tumor cell clone or two clones. This requires customizing specific probes of GCA genes and the EML4 gene to explain this problem, which is time-consuming and expensive. We will further study in future work.

Furthermore, despite surgery, the 5-year survival rates of stage III NSCLC are low and most patients mainly die from metastatic disease (13, 14). Thus, our patient diagnosed as stage IIIB-N2 ALK-positive NSCLC had a great risk of disease recurrence. We administrated alectinib as adjuvant targeted therapy for our case, leading to 20 months of DFS to date which has been longer than approximately 14.1 months of median survival time of IIIB NSCLC patients (14). From the perspective of clinical practice, we believed that the use of alectinib adjuvant targeted therapy for this patient with this novel non-reciprocal ALK-GCA (A19: intragenic) and EML4-ALK (E20: A20) may be effective. Furthermore, the previous study reported that the 5′-ALK (GCA-ALK) of non-reciprocal ALK fusion would affect the efficacy of ALK-TKI (10). Therefore, it cannot be said that the response to alectinib is totally due to EML4-ALK fusion. A clinical trial ALINA investigating the role of adjuvant ALK-TKI in locally advanced ALK+NSCLC currently is still ongoing (15), and adjuvant alectinib may be promising. Furthermore, some research suggested that N2-NSCLC patients benefit from PORT (16, 17). Our patient also received PORT as adjuvant treatment after surgery, without serious side effects when combining PORT and target adjuvant. Hence, it may provide a rationale to investigate the efficacy and safety of the ALK-TKI target adjuvant combined with PORT further for IIIB-N2 locally advanced ALK+ NSCLC patients for longer survival. Finally, we speculated that this novel non-reciprocal ALK-GCA (A19: intragenic) and EML4-ALK (E20: A20) in the patient may be sensitive to alectinib.

## Conclusion

We identified a novel non-reciprocal ALK rearrangement, ALK-GCA (A19: intragenic) and EML4-ALK (E20: A20), by using powerful NGS, expanding the spectrum of ALK fusion in ALK+ NSCLC. In addition, this non-reciprocal fusion may be sensitive to alectinib treatment.

## Data Availability Statement

The original contributions presented in the study are included in the article/**Supplementary Material**. Further inquiries can be directed to the corresponding authors.

## Ethics Statement

The studies involving human participants were reviewed and approved by Institutional Review Board of West China Hospital, Sichuan University. The patients/participants provided their written informed consent to participate in this study.

## Author Contributions

XZ and QW wrote the original draft and prepared the figures and reviewed the literature. DP and LY collected the data and edited the manuscript. WW Pathologic data collection and analysis. DZ supervised, provided the resource, and reviewed the article. FX conceived the idea and reviewed the article. All authors contributed to the article and approved the submitted version.

## Funding

This work was supported by the National Natural Science Foundation of China (81573024) which will pay for open-access publication fees.

## Conflict of Interest

The authors declare that the research was conducted in the absence of any commercial or financial relationships that could be construed as a potential conflict of interest.

## Publisher’s Note

All claims expressed in this article are solely those of the authors and do not necessarily represent those of their affiliated organizations, or those of the publisher, the editors and the reviewers. Any product that may be evaluated in this article, or claim that may be made by its manufacturer, is not guaranteed or endorsed by the publisher.
