# Supplementary material for: Case Report: A Novel Non-Reciprocal ALK Fusion: ALK-GCA and EML4-ALK Were Identified in Lung Adenocarcinoma, Which May Respond to Alectinib Adjuvant-Targeted Therapy
Source: Front Oncol. 2022 Jan 5;11:782682. doi: 10.3389/fonc.2021.782682 (PMC8767047; doi:10.3389/fonc.2021.782682)
Supplement: Supplementary file 1 [file DataSheet_1.pdf]

68 Lung Cancer-Related Gene List

AKT	ALK	APC	AR	ARAF
ATM	AXT	BCL2L11	BRAF	BRCA1
BRCA2	CCND1	CD74	CDK4	CDK6
CDKN2A	CTNNB1	CYP2D6	DDR2	DPYD
EGFR	ERBB2	ERBB3	ERBB4	ESR1
FGF19	FGF3	FGF4	FGFR1	FGFR2
FGFR3	FLT3	HRAS	IDH1	IDH2
IGF1R	JAK1	JAK2	KDR	KIT
KRAS	MAP2K1	MET	MTOR	MYC
NF1	NOTCH1	NRAS	NRG1	NTRK1
NTRK2	NTRK3	PDGFRA	PIK3CA	PTCH1
PTEN	RAF1	RB1	RET	ROS1
SMAD4	SMO	STK11	TOP2A	TP53
TSC1	TSC2	UCT1A1		

Fusion Gene List

ALK	FGFR1	FGFR3	NRG1	NTRK1
RET	ROS1			
