# Supplementary figures and images for: Case Report: A Novel Non-Reciprocal ALK Fusion: ALK-GCA and EML4-ALK Were Identified in Lung Adenocarcinoma, Which May Respond to Alectinib Adjuvant-Targeted Therapy
Source: Front Oncol. 2022 Jan 5;11:782682. doi: 10.3389/fonc.2021.782682 (PMC8767047; doi:10.3389/fonc.2021.782682)

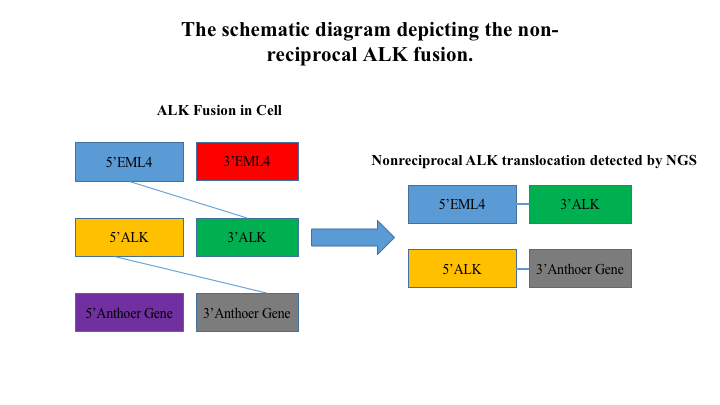

Supplement: Supplementary file 2 [file Image_1.tiff]
